# External fixation compared to intramedullary nailing of tibial fractures in the rat

**DOI:** 10.3109/17453670903035567

**Published:** 2009-06-01

**Authors:** Ulf E W Sigurdsen, Olav Reikeras, Stein Erik Utvag

**Affiliations:** ^1^Department of Orthopedic Surgery, Faculty Division Akershus University Hospital, University of OsloNorway; ^2^Institute of Surgical Research, Rikshospitalet Medical CenterOsloNorway; ^3^Department of Orthopedic Surgery, Rikshospitalet Medical CenterOsloNorway; ^4^Department of Orthopedic Surgery, Akershus University HospitalOsloNorway

## Abstract

**Background and purpose** It is not known whether there is a difference in bone healing after external fixation and after intramedullary nailing. We therefore compared fracture healing in rats after these two procedures.

**Methods** 40 male rats were subjected to a standardized tibial shaft osteotomy and were randomly assigned to 2 treatment groups: external fixation or intramedullary nailing. Evaluation of half of each treatment group at 30 days and the remaining half at 60 days included radiography, dual energy radiographic absorbtiometry, and mechanical testing.

**Results** Radiographically, both treatment groups showed sign of fracture healing with gradual bridging of the fracture line, while with intramedullary nailing the visible collar of callus was increased peripherally, indicative of periosteal healing. At 30 days, densitometric and mechanical properties were similar in the 2 groups. At 60 days, however, the intramedullary nailed bones had more strength, greater callus area, and higher bone mineral content in the callus segment compared to externally fixated fractures.

**Interpretation** Tibial shaft fractures in the rat treated with external fixation and intramedullary nailing show a similar healing pattern in the early phase of fracture healing, while at the time of healing intramedullary nailing provides improved densitometric properties and superior mechanical properties compared to external fixation. Clinical findings indicate that intramedullary nailing in human tibial fractures may be more advantageous for bone healing than external fixation, in a similar way.

## Introduction

Over the past few decades, different surgical techniques have been used for stabilization of tibial fractures, mainly internal fixation by intramedullary nailing with or without reaming or external fixation.

The external fixator appeared in the mid-19th century and was refined and improved in 1938 by Hoffmann (Vidal 1983). Despite well-known disadvantages such as the need for patient compliance and the risk of pin tract complications, it is the preferred option in many complicated tibial fractures, and tibial fractures are now the prime area of application of external fixation (Rüedi et al. 2007). In cases of unstable fractures and in multitrauma patients with increased inflammatory response, it is the preferred initial treatment according to the recent concept of damage-control orthopedics (Hildebrand et al. 2004). Recent advances in biomechanics and biomaterials have resulted in improvements in external fixation frames, and they can now remain in place for prolonged periods of time without degradation of the pin-bone surface (Watson 2006).

The classic tight-fitting Küntscher nail, which is implanted intramedullary after reaming, gives good cortical contact, stability, and protection against bending and shear forces. However, it is rather inefficient against torque and is unable to prevent axial shortening. Nail design has progressed, and locked nails provide better torsional and axial stability. Clinical advantages include high patient acceptance, access for soft tissue care, and secure control of alignment and rotation. Disruption of intramedullary bone circulation has been a concern, especially with reaming (Koval et al. 1991). This concern is less pronounced with the development of the unreamed locked intramedullary nail (Schemitsch et al. 1994, Forster et al. 2005, Petrisor et al. 2005). Locked intramedullary nailing is now the standard treatment for uncomplicated tibial fractures.

It has never been shown which of these two treatment options is better with regard to bone healing. We therefore performed an experimental study in rats to compare bone healing in external fixation and intramedullary nailing of tibial shaft fractures.

## Methods

### Animals and surgical procedures

40 male Wistar rats (Møllegårds Avlslaboratorium, Eiby, Denmark) weighing 306–378 g were used. The animals were housed in rodent cages with 2 rats in each cage, and received a standard rodent diet (RM3(E); Special Diets Services, Witham, UK). The dark-light cycle was 12 h/12 h. The experiment conformed to the Norwegian Council of Animal Research Code for the Care and Use of Animals for Experimental Purposes.

Peroperative anesthesia was 0.03 mL/kg of a working solution of fentanyl/fluanisone and midazolam administered subcutaneously. The left tibia was exposed through a 20-mm anterior incision from the tuberositas tibia and in distal direction. The muscles on the medial and lateral aspect of the tibia were elevated from the tibia and the anterior two-thirds of the bone was cut with a fine-tooth circular saw blade mounted on an electric drill at the level of the anterior ridge. The remaining one-third was then broken manually, leaving the fibula intact.

**Figure 1.**
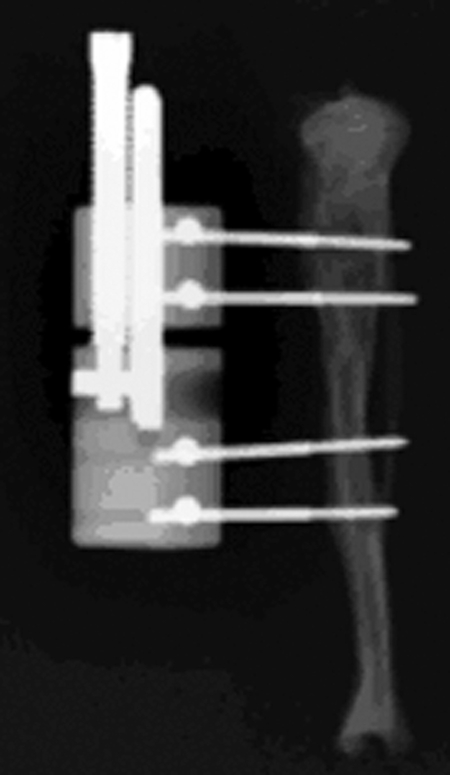
Rat tibial diaphyseal fracture 60 days after external fixation.

**Figure 2.**
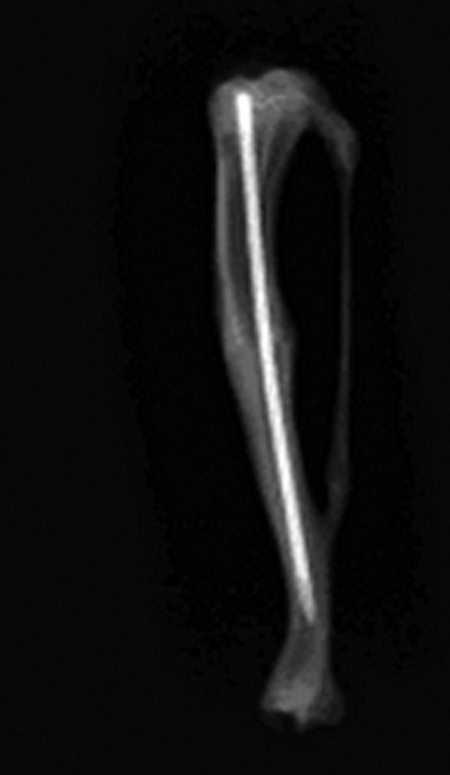
Rat tibial diaphyseal fracture 60 days after intramedullary nailing.

The rats were randomly assigned to external fixation (n = 20) or intramedullary nailing (n = 20). The external fixator has been described previously (Mark et al. 2003) (Figure 1). 2 pins (1.0 mm in diameter) were inserted proximal to the fracture and 2 distal. The core drill-holes in the tibia were 0.8 mm. Fixator-bone offset was 6 mm. The external fixators were placed anterolaterally, giving the animals freedom of physical movement. The nails were inserted from the proximal side into the bone marrow cavity through the anterior tip of the tibial plateau to the distal tibiofibular junction, with the knee in a flexed position (Figure 2). The nails were cut flush to the bony surface at the insertion side. There was no reaming or locking of the nail. The medial and posterior segments were left attached to the bone. The operation wound was closed in 2 layers, with absorbable suture. A layer of transparent film dressing was then sprayed on the sutured wound.

The alignment and accurate reduction of the fracture were verified perioperatively both visually and manually. The animals were observed daily for the first 3 days and then on a weekly basis. Buprenorphine, 0.05 mg/kg, was injected subcutaneously twice daily on the first 3 postoperative days.

The rats were killed with an intraperitoneal injection of 100 mg/kg pentobarbital. Half of each treatment group was killed and evaluated at 30 days and the remaining half at 60 days. As a result, there were 4 groups for statistical analysis with 10 animals in each. The tibias were dissected and examined visually and radiographically before nails and external devices were removed. Between dissection and radiographic, densitometric, and mechanical examination, the bones were kept frozen at –80ºC.

### Bone evaluations

Radiographs were taken on a standard clinical digital system. Focus-to-film distance was 115 cm and X-ray tube settings were 46 kV and 1.0 mAs.

Dual-energy radiographic absorptiometry (DXA) was performed using a densitometer system for research animals (Piximus; Lunar Corp, Madison, WI). The X-ray tube voltage was 80 kV with a current of 400µA and a focal spot size of 0.25 mm × 0.25 mm. Focal spot-to-image receptor distance was 32 cm. The values for callus area (CA), bone mineral density (BMD), and bone mineral content (BMC) were automatically analyzed and calculated by the software from a 3.8-mm region of interest at the fracture site.

The tibias were placed in-between gauze pads that were wet with normal saline before a bending test was performed using a universal testing machine with a servo-hydraulic mechanical linear drive actuator with 100 mm of total vertical displacement and maximum axial tension loading of 250 N (MTS 858 Mini Bionix; MTS Systems Corp., Eden Prairie, MN). The setup included a 3-point cantilever bending test designed to test the fracture site and a standard program setting the vertical travel speed to 160 mm/min. The log file was then converted to a classic stress-strain curve, and values for the basic mechanical properties strength, rigidity, and work to fracture were obtained using a mathematical software package (Origin version 7.5; OriginLab Corp., Northampton, MA). A similar bending test performed on cadaver tibia-implant constructions for external fixation (n = 5) and intramedullary nailing (n = 5) showed mean rigidity of 3.2 (SE 0.6) N/mm and 0.6 (0.1) N/mm, respectively. In comparison, the mean rigidity of intact tibia (n = 5) was 3.6 (0.3) N/mm.

### Statistics

A detection power of 80% for a clinically interesting difference in fracture strength of 10 N (SD 7), based on results from a previous study (Utvag et al. 2003), with a level of significance of 0.05, required at least 9 animals in each group. An independent sample-t-test was used to test for statistically significant differences.

## Results

The animals resumed normal function within a few days. Of the 20 rats treated with external fixation, 2 rats—one with a pin fracture and one with loosening of the external fixator—were excluded from the study.

In both external fixation and intramedullary nailing, the fracture line was less visible at 60 days than at 30 days. In the external fixation group, the radiographs showed less visible external callus at both 30 and 60 days compared to the intramedullary nailing (Table 1). Radiographs of intramedullary nailed group showed periosteal healing with more visible external callus formation at both 30 and 60 days.

**Table 1.** Callus area (mm^2^), bone mineral content (BMC, 10^-3^ g) and bone mineral density (BMD, 10^-3^ g/cm^2^) in 3.8-mm region of interest at fracture sites in tibial diaphyseal fractures in rats at 30 and 60 days following treatment with external fixation and intramedullary nailing. Mean (SE)

	External fixation	Intramedullary nailing	p-value (t-test)
Callus area (mm^2^)			
30 days	24 (1.3)	24 (0.7)	0.6
60 days	19 (0.6)	23 (0.7)	0.002
BMC (10^-3^ g)			
30 days	46 (3.2)	51 (2.6)	0.2
60 days	37 (2.3)	48 (2.8)	0.009
BMD (10^-3^ g/cm^2^)			
30 days	20 (1.0)	21 (1.0)	0.3
60 days	19 (0.9)	22 (1.1)	0.2

The bone mineral density was higher in the intramedullary nailed group than in the external fixation group at 30 and 60 days, but the difference was not statistically significant. Also, bone mineral content and callus area were higher in the animals with intramedullary nailing, at both 30 and 60 days, compared to those with external fixation, and the differences were statistically significant at 60 days (p = 0.009 and p = 0.002, respectively).

The fractures treated with intramedullary nailing gained a higher degree of mechanical strength and fracture energy at 60 days than the fractures that were externally fixated (p = 0.02 and p = 0.008, respectively) (Table 2).

**Table 2.** Bending strength (N), energy (N × mm), and rigidity (N/mm) in tibial diaphyseal fractures in rats at 30 and 60 days following treatment with external fixation and intramedullary nailing. Mean (SE)

	External fixation	Intramedullary nailing	p-value (t-test)
Strength (N)			
30 days	11 (3.1)	17 (2.6)	0.2
60 days	19 (2.8)	30 (3.1)	0.02
Energy (N × mm)			
30 days	25 (6.8)	37 (8.2)	0.3
60 days	32 (3.9)	73 (13)	0.008
Rigidity (N / mm)			
30 days	6 (2.7)	5 (0.4)	0.6
60 days	6 (0.9)	6 (0.6)	0.7

## Discussion

We found that in the early phase of fracture healing, externally fixated and intramedullary nailed fractures of the tibia underwent mineralization and gained mechanical characteristics of the callus segment to similar extents. In contrast, at 60 days, the fractures treated with intramedullary nailing had a larger callus area and bone mineral content at the fracture site. More importantly, they proved to be stronger with higher fracture energy when mechanically tested.

Clinical studies comparing surgical techniques in fracture treatment are hampered by the heterogenity of fractures, and the evaluation of mechanical properties of bone healing are limited to qualitative or semi-quantitative surrogate markers such as time to weight bearing, radiographical union, reoperations, and number of refractures. Several quantitative imaging techniques have been tested as predictors of mechanical properties of healing bone, but no single, clinically applicable parameter has been found. Thus, animal experiments with standardized osteotomies and direct mechanical testing of every treated bone (to failure) is necessary in order to obtain an objective evaluation of fracture healing under different conditions.

In our study, we did not radiograph the bones primarily, as we had visual control of bone adaptation and alignment. Radiographic evaluation of the bones was done after killing, but this could not be done in a true blind fashion because of the clearly visible external fixator pins/pin holes. This may represent some bias. However, the mechanical bending test gave an objective and unbiased evaluation of healing.

Caution must be exercised when applying the results of any animal study to human clinical practice, or to explain or support clinical findings. Biological differences exist; human bones have a haversian lamellar structure and rat bones do not. There may be species-specific reactions. However, several features of rat bones are similar to those of human bones in different parts of the skeleton, specifically the basic physiological remodeling mechanisms. Also, the deformation and biological repair of long bones are fairly constant across species (Liebschner 2004). The rat is now recognized as a suitable model for skeletal research relevant to humans and widely used 3060.

Early studies indicated that leg fractures in the rat regain mechanical properties similar to those of intact bone at 60 days (Ekeland et al. 1982). In our study, fracture strength at 60 days in the intramedullary nailed group was 85% of that of intact bone, and the corresponding figure was 54% in the externally fixated group. As we also wanted information about the medium-term healing process, we evaluated differences in mechanical properties and mineralization at 30 days and at 60 days.

In experimental studies, it has been shown that the rotational stability provided by an intact fibula favors healing (Horn et al. 2008, Klein et al. 2004). The fibula is often fractured in human tibial fractures, and compensated for by interlocking of the nail. All fibulas were left intact in our study for rotational stability. Thus, our experimental situation and the usual clinical setting differ, but the significance of this is unclear.

Fracture healing is a unique biological repair process that is influenced by the mechanical milieu; we have known this for over half a century (Yamagishi and Yoshimura 1955). An increased interfragmentary gap or movement may result in malunion, delayed union or even nonunion. Flexible fixation promotes some motion at the fracture site in favor of secondary bone healing with the characteristic development of bridging periosteal callus until cortical healing occurs. While it is well accepted that interfragmentary motion influences callus formation and the healing of fractures in both intramedullary nailing and external fixation, it remains unclear which biomechanical conditions are optimal for the fracture healing process (Smith-Adaline et al. 2004, Jagodzinski and Krettek 2007). In our study, the length of incision and surgical manipulation of the soft tissue around the fracture site may have been slightly more extensive in the external fixation procedure than in intramedullary nailing even though we aimed at similar soft tissue dissection in the surgical protocol. This might have reduced circulation and inhibited healing, and partly explain the lower amount of callus formation and inferior mechanical test results in the external fixation group.

Major soft tissue injuries delay bone healing in tibial fractures (Gustilo et al. 1984). In our study, the length of incision and surgical manipulation of the soft tissue around the fracture site may have been slightly more extensive in the external fixation procedure than with intramedullary nailing, even though in the surgical protocol we had aimed at similar soft tissue dissection in the treatment groups. This may have damaged the circulation and inhibited healing to some extent, and partly explains the lower amount of callus formation and the inferior mechanical testing results than for intramedullary nailing. In clinical practice, application of external fixators probably means less soft tissue damage.

Our results support intramedullary nailing as the standard treatment for tibial fractures in humans. In a prospective, randomized study of unstable tibial fractures, 78 patients were distributed into 2 treatment groups to be treated with either external fixation (n = 41) or intramedullary nailing (n = 38), excluding patients with soft tissue problems of importance (Braten et al. 2005). The results of treatment were similar, but there were more reoperations with external fixation. Also, the intramedullary nailing group had earlier unprotected weight bearing (12 weeks versus 20 weeks; p < 0.001).

Clinical studies suggest that when initial external fixation is used in complicated tibial shaft fractures, a conversion to intramedullary nailing must follow as soon as possible, usually within 2 weeks (Antich-Adrover et al. 1997, Henley et al. 1998, Della Rocca and Crist 2006). The rationale for performing the conversion has been that intramedullary nailing may provide a better mechanical milieu in which bone healing is enhanced, ultimately producing superior mechanical properties without the risk of pin tract infection. Our evaluation at 30 days revealed similar healing for the 2 implant types, but at 60 days the results of intramedullary nailing were preferable to those of bone healing. Thus, exchange intramedullary nailing after initial external fixation may enhance the bone repair process.

In conclusion, our study suggests that in tibial diaphyseal fractures, treatment with external fixation is equivalent to intramedullary nailing in terms of regain of mechanical properties in the initial phase of healing. However, at the time of healing, intramedullary nailing provides significantly higher callus maturation (measured as mineralization of the callus segment) and results in superior mechanical properties compared to external fixation. Clinical findings indicate that compared to external fixation, intramedullary nailing in human tibial fractures may be advantageous for bone healing.
